# Characterization of Changes in Global Genes Expression in the Distal Colon of Loperamide-Induced Constipation SD Rats in Response to the Laxative Effects of *Liriope platyphylla*

**DOI:** 10.1371/journal.pone.0129664

**Published:** 2015-07-07

**Authors:** Ji Eun Kim, So Hae Park, Moon Hwa Kwak, Jun Go, Eun Kyoung Koh, Sung Hwa Song, Ji Eun Sung, Hee Seob Lee, Jin Tae Hong, Dae Youn Hwang

**Affiliations:** 1 Department of Biomaterials Science, College of Natural Resources & Life Science/Life and Industry Convergence Research Institute, Pusan National University, Miryang, 627–706, Korea; 2 Department of Food Science and Nutrition, College of Human Ecology, Pusan National University, Busan, 609–735, Korea; 3 College of Pharmacy and Medical Research Center, Chungbuk National University, Chungju, 361–763, Korea; University of Nevada School of Medicine, UNITED STATES

## Abstract

To characterize the changes in global gene expression in the distal colon of constipated SD rats in response to the laxative effects of aqueous extracts of *Liriope platyphylla* (AEtLP), including isoflavone, saponin, oligosaccharide, succinic acid and hydroxyproline, the total RNA extracted from the distal colon of AEtLP-treated constipation rats was hybridized to oligonucleotide microarrays. The AEtLP treated rats showed an increase in the number of stools, mucosa thickness, flat luminal surface thickness, mucin secretion, and crypt number. Overall, compared to the controls, 581 genes were up-regulated and 216 genes were down-regulated by the constipation induced by loperamide in the constipated rats. After the AEtLP treatment, 67 genes were up-regulated and 421 genes were down-regulated. Among the transcripts up-regulated by constipation, 89 were significantly down-regulated and 22 were recovered to the normal levels by the AEtLP treatment. The major genes in the down-regulated categories included Slc9a5, klk10, Fgf15, and Alpi, whereas the major genes in the recovered categories were Cyp2b2, Ace, G6pc, and Setbp1. On the other hand, after the AEtLP treatment, ten of these genes down-regulated by constipation were up-regulated significantly and five were recovered to the normal levels. The major genes in the up-regulated categories included Serpina3n, Lcn2 and Slc5a8, whereas the major genes in the recovered categories were Tmem45a, Rerg and Rgc32. These results indicate that several gene functional groups and individual genes as constipation biomarkers respond to an AEtLP treatment in constipated model rats.

## Introduction

Constipation is a chronic gastrointestinal disorder that exhibits symptoms, such as infrequent bowel movements, difficulties during defecation, and the sensation of incomplete bowel evacuation [1,2,3]. This disease is often caused by insufficient dietary fiber intake, inadequate fluid intake, decreased physical activity, medication side effects, hypothyroidism, and obstructions by colorectal cancer in adults [4]. To date, a range of chemical drugs including senna, correctol, exlax, senokot, and gaviscon, are commonly used for the treatment of constipation; however, their use is limited due to their high cost and undesirable side effects [5,6]. In addition, some promotility agents, including cisapride and tegaserod, are used widely for the treatment of constipation, even though they cause cardiac arrhythmia and coronary artery contraction [7,8]. Furthermore, some plant extracts, such as extracts of *Aloe ferox Mill*., ethanol extracts of agarwood (*Aquilaria sinensis*, *Aquilaria crasna*) leaves and *Ficus carica* paste, exhibit laxative properties based on their ability to increase the intestinal motility, frequency and weight of stools, and ileum tension [9,10].

Recently, several herbal medicines have received increased attention as novel therapeutic drugs for the treatment of constipation. Among these, *L*. *platyphylla* containing saponin, isoflavone, oligosaccharide, and succinic acid have long been used for the treatment of asthma as well as bronchial and lung inflammation [11,12]. *L*. *platyphylla* is known to potently inhibit airway inflammation and hyper-responsiveness in a murine model of asthma by modulating the relationship between Th1/Th2 cytokine imbalance [12], atopic dermatitis induced by phthalic anhydride treatment [13,14], and act effectively on the process of obesity and diabetes [15,16]. In addition, significant alteration of nerve growth factor (NGF) secretion as well as its related signaling pathway has been observed in the extracts and compounds isolated from *L*. *platyphylla*-treated PC12 cells [17,18]. In particular, *L*. *platyphylla* induced laxative effects including an enhancement of the stool weight, urine excretion, villus length, crypt layer, mucin secretion, and lipid droplet secretions [19]. Nevertheless, there are no reports of the genes regulated by an AEtLP treatment during constipation induced by a loperamide treatment in SD rats.

This study characterized the changes in global gene expression in the distal colon of constipated model rats in response to the laxative effects of AEtLP.

## Results

### Composition and functional components of L. platyphylla

**Fig 1:**
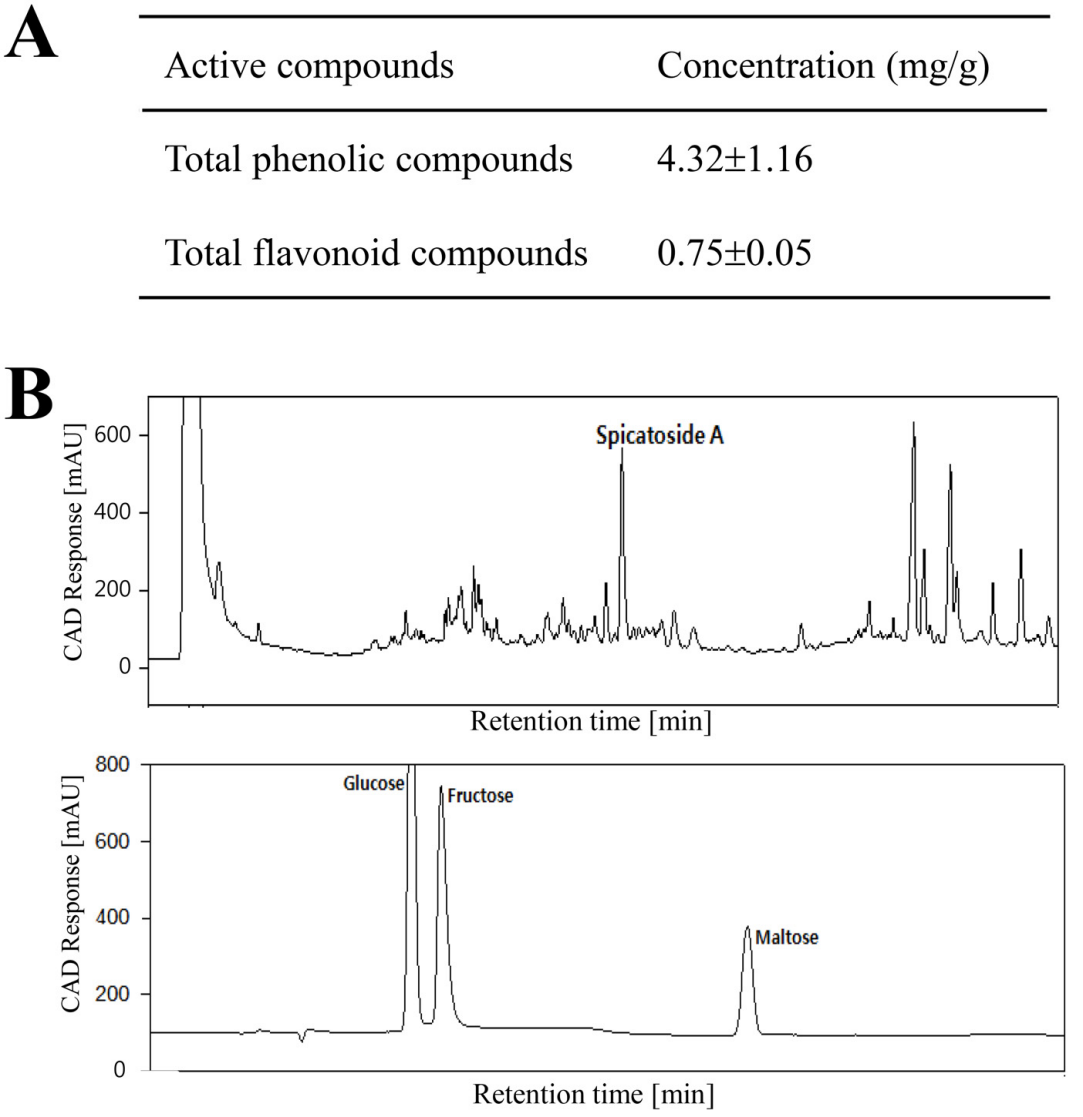
Functional components of LP. **(A) The concentration of total phenolic compounds and flavonoid compounds was measured, as described in the Materials and Methods.** (B) Spicatoside A, glucose, fructose and maltose in LP were detected at the corresponding retention times in the HPLC chromatogram.

As shown Fig 1, the roots of *L*. *platyphylla* contain high concentrations of several bioactive components related to the laxative effects. The concentration of total phenolic compounds and total flavonoid compounds were 4.32 mg/g and 0.75 mg/g, respectively. In addition, a peak indicating high level of spicatoside A as well as some sugars was detected in the LP at an appropriate retention time by high performance liquid chromatography (HPLC) analysis.

### Identification of the laxative effects in constipated SD rats

**Fig 2:**
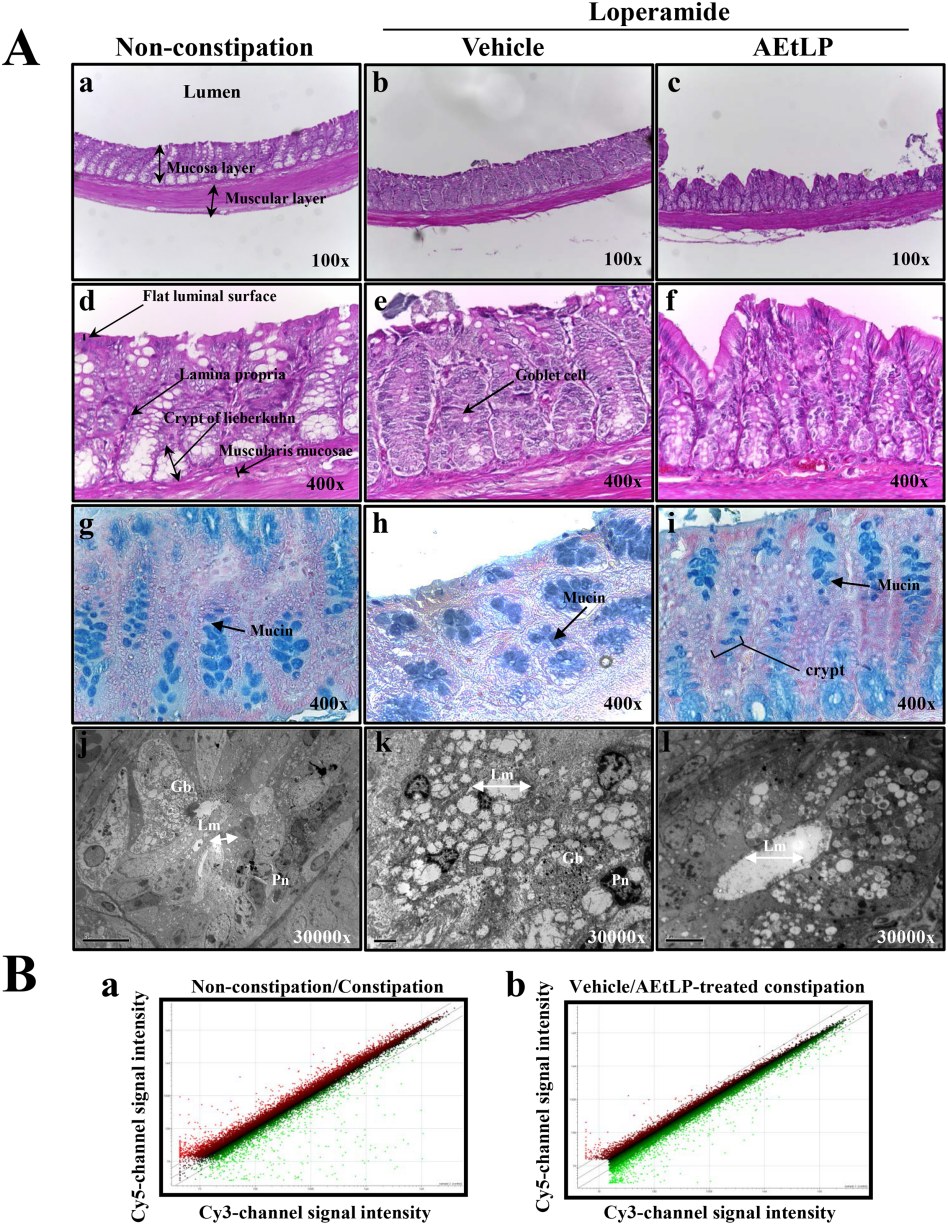
Effects of AEtLP on the histological parameters and global gene expression in loperamide-induced constipated rats. (A) H&E or Alcian blue-stained sections and ultra-thin sections of the distal colon in the non-constipation group (Aa, d, g and j), vehicle-treated constipation group (Ab, e, h and k), or AEtLP-treated constipation group (Ac, f, i and l) were observed by optical microscopy and TEM at the indicated magnifications. (B) Scatter plots. Each point represents the average expression for the same gene from the non-constipation/constipation group (Ba) and vehicle-/AEtLP-treated constipation group. (Bb) The Cy3-channel indicates the control sample and the Cy5-channel indicates the test sample. The Cy5-channel axis shows that the probe of the Cy5-channel (vehicle-treated constipation group or AEtLP-treated constipation group) shows a signal intensity more than two times higher than that of the Cy3-channel (non-constipation group or constipation group), whereas the Cy3-channel axis shows the reverse situation.

The administration of loperamide successfully induced constipation, as indicated by the decreased stool number, stool weight, water contents of the stool, and urine volume in SD rats. On the other hand, these levels were almost recovered to those in the non-constipation group in response to the AEtLP treatment, even though the type of stool was maintained in their original form as hard feces (Table 1). Furthermore, after the AEtLP treatment, the mucosa thickness, the average muscle thickness, flat luminal surface thickness, mucin production, number of goblet cells and number of lieberkuhn crypts increased by more than 10.9–190% compared to the vehicle-treated constipation group, while the expression of muscarinic acetylcholine receptor (mAchR) decreased with 7.7–25%. Most histological alterations and enhancement of mAchR expression in the distal colon were mostly recovered to these of the Non-constipation group, even though there were small differences between them. (Fig 2A and Table 2). These results show that AEtLP may induce smoothing of the hard stool in constipation through the functional and histological alterations of the distal colon in the constipated SD rats induced by a loperimide treatment and may induce changes in the regulation of gene expression.

**Table 1:** Laxative effect of AEtLP on the excretion parameter of constipated SD rats.

	Non-constipation	Constipation	
		Vehicle	AEtLP
Food intake (g/day)	23.94±0.18	11.85±0.72^a^	10.22±0.98 ^a^
Water consumption (ml/day)	4.54±0.08	4.77±0.46	4.09±0.22
Number of stools (ea)	28.71±8.95	14.33±4.35 ^a^	26.58±8.67^b^
Weight of stools (g)	4.59±0.48	2.88±0.79 ^a^	5.54±0.64 ^b^
Water contents of stools (%)	46.25±8.77	23.54±5.11 ^a^	48.79±7.57 ^b^
Volume of urine (ml/day)	13.28±1.61	9.48±1.21 ^a^	12.15±1.12 ^b^
Types of stool	Hard feces	Hard feces	Hard feces

^a^, *P*<0.05 is the significant level compared with non-constipation group.

^b^, *P*<0.05 is the significant level compared with the vehicle-treated constipation group.

**Table 2:** Alteration of the histological parameter and mAchRs expression of the constipated SD rats.

Categories	Non-constipation	Constipation	
		Vehicle	AEtLP
Mucosa thickness (μm)	183.75±7.09	164.44±8.84^a^	182.15±14.68^b^
Muscle thickness (μm)	154.99±14.45	65.33±11.74^a^	106.51±21.45^a^ ^,^ ^b^
Flat luminal surface thickness (μm)	16.25±2.03	10.13±1.87^a^	29.56±3.54^a^ ^,^ ^b^
Number of goblet cell (ea)	104.67±7.02	29.00±4.58^a^	73.33±10.21^a^ ^,^ ^b^
Number of crypt of lieberkuhn (ea)	20.33±2.52	9.67±1.53^a^	17.33±3.06^b^
Relative expression level of mAchR2	1±0.12	1.6±0.2^a^	1.2±0.18^b^
Relative expression level of mAchR3	1±0.11	1.3±0.14^a^	1.2±0.13^a^ ^,^ ^b^

^a^, *P*<0.05 is the significant level compared with non-constipation group.

^b^, *P*<0.05 is the significant level compared with the vehicle-treated constipation group.

### Filtering and selection for differentially-expressed genes

As shown Fig 2B, a considerable number of genes are also expressed at elevated or at reduced levels in the distal colon of the constipation group relative to the non-constipation group, even though the expression levels of most genes showed no difference. Compared to the non-constipation SD rats, a total of 20,020 genes were detected as differentially expressed genes in the constipation SD rats. Further analysis revealed 797 up-regulated and down-regulated genes in the constipation rats compared to the non-constipation rats (Table 3A). In addition, a total of 20,331 genes were found to be expressed differentially in the AEtLP-treated constipation rats compared to the vehicle-treated constipation rats. Additional analysis revealed 488 up-regulated or down-regulated genes in the AEtLP-treated constipation rats (Table 3A).

**Table 3:** Selection of the differentially expressed genes.

A, Differentially expressed gene in loperamide-induced constipation rats.			
Categories	Number of transcripts		
Total	30,367		
Filtered gene	20,020		
|fold|≥ 2	797	Up	581
		Down	216
B, Differentially expressed gene in loperamide-induced constipation rats after AEtLP treatment			
Categories	Number of transcripts		
Total	30,367		
Filtered gene	20,331		
|fold|≥ 2	488	Up	67
		Down	421

### Ontology categories of constipation-regulated gene expression

PANTHER/X ontology identified 15 GO categories showing significantly elevated expression in the list of up-regulated transcripts as well as 14 GO categories showing markedly decreased expression in the list of down-regulated transcripts (Table 4). Furthermore, the ontology classification of AEtLP-regulated gene expression was categorized in the distal colon of the AEtLP treated rats. Fourteen GO categories showed significantly elevated expression in the list of the up-regulated transcripts, whereas 16 markedly overexpressed GO categories were identified in the list of the down-regulated transcripts (Table 5).

**Table 4:** Gene ontology categories in the loperamide-induced constipation rats.

Category Term	Count
Up-regulated genes (15 categories)	
Signal transduction	118
Cell differentiation	89
Transcription	62
Apoptosis	46
Cell proliferation	46
Immune response	45
Regulation of cellular protein metabolic process	42
Neurogenesis	31
Inflammatory response	19
Cell cycle	18
Response to oxidative stress	17
Aging	17
Angiogenesis	15
Extracellular matrix	13
DNA repair	3
Total	581
Down-regulated genes (14 categories)	
Signal transduction	36
Cell proliferation	31
Cell differentiation	22
Transcription	22
Apoptosis	20
Immune response	14
Inflammatory response	14
Regulation of cellular protein metabolic process	11
Extracellular matrix	11
Neurogenesis	9
Response to oxidative stress	9
Angiogenesis	7
Cell cycle	6
Aging	4
Total	216

**Table 5:** Gene ontology categories in loperamide-induced constipation rats after AEtLP treatment.

Category Term	Count
Up-regulated genes (14 categories)	
Signal transduction	17
Cell differentiation	8
Cell proliferation	7
Regulation of cellular protein metabolic process	5
Apoptosis	5
Cell cycle	4
Transcription	4
Extracellular matrix	3
Neurogenesis	3
Angiogenesis	3
Immune response	3
Aging	2
Inflammatory response	2
Response to oxidative stress	1
Total	67
Down-regulated genes (16 categories)	
Signal transduction	76
Cell differentiation	67
Immune response	47
Apoptosis	44
Cell proliferation	38
Transcription	38
Regulation of cellular protein metabolic process	27
Neurogenesis	19
Cell cycle	13
Inflammatory response	13
Aging	10
Response to oxidative stress	9
Angiogenesis	7
Extracellular matrix	6
RNA splicing	5
DNA repair	2
Total	421

### Genes simultaneously altered by loperamide and AEtLP treatment

The genes recovered by the AEtLP treatment among those regulated by constipation based on a 2-fold change in expression were selected from the total transcripts of the distal colon. Among the up-regulated genes, 89 transcripts were down-regulated by more than 2-fold after the AEtLP treatment, whereas 22 were recovered to the normal levels. In addition, a total of 15 down-regulated genes could be classified as either up-regulated or recovered to the normal levels (Table 6). Overall, these results suggest that constipation and laxative effects are more closely related to the up-regulated genes than the down-regulated genes.

**Table 6:** Up or down regulated genes in the loperamide-induced constipation rats after the AEtLP treatment at the level of significance.

Loperamide	AEtLP	Number of transcripts
Up regulation	Down regulation to above 2 folds	89
	Recover to normal level*	22
Down regulation	Up regulation to below 2 folds	10
	Recover to normal level*	5

*The values of fold change are between 0.5< and <1.5.

### Genes showing the greatest up-regulation and down-regulation

To characterize the genes down-regulated by the AEtLP treatment after overexpression induced by constipation, those that showed at least a 2-fold change were selected, resulting in 89 down-regulated genes and 22 recovered genes. Of the 89 down-regulated genes, the largest difference was observed in Slc9a5 (11.6-fold), which was identified as a sodium ion transport-related gene, followed by Slc5a4a, Klk10, Fgf15, Alpi, and Chmp4bl1 (Table 7). In addition, 22 down-regulated genes including Cyp2b2, Ace, Cyp2j4, G6pc, Setbp1, and Nphs1 were recovered to the normal level after the AEtLP treatment (Table 8).

**Table 7:** List of genes (19)* down-regulated by the AEtLP treatment among total genes up-regulated by constipation.

GeneSymbol	GeneName	Accession No.	Fold of change in vehicle-treated group	Fold of change in AEtLP-treated group	GO category
Slc9a5	solute carrier family 9 (sodium/hydrogen exchanger), member 5	NM_138858	11.60	0.49	Cellular component, cation transport, sodium ion transport
Slc5a4a	solute carrier family 5, member 4a	NM_001106383	11.47	0.47	transporter activity, ion transport, sodium ion transport
Klk10	kallikrein related-peptidase 10	NM_001004100	8.61	0.40	Molecular function, catalytic activity
Fgf15	fibroblast growth factor 15	NM_130753	6.79	0.35	neural crest cell migration, fibroblast growth factor receptor binding
Alpi	alkaline phosphatase, intestinal	NM_022665	6.21	0.41	magnesium ion binding
Chmp4bl1	chromatin modifying protein 4B-like 1	XM_002726346	5.41	0.26	protein transport
Aldob	aldolase B, fructose-bisphosphate	NM_012496	4.72	0.18	liver development
Sphk1	sphingosine kinase 1	NM_133386	4.63	0.47	magnesium ion binding, blood vessel development
Cyp2b2	cytochrome P450, family 2, subfamily b, polypeptide 2	NM_001198676	4.51	0.47	monooxygenase activity, endoplasmic reticulum
Btnl7	butyrophilin-like 7	NM_212488	4.30	0.40	membrane
Setbp1	SET binding protein 1	XM_001056790	3.96	0.38	DNA binding
Sst	somatostatin	NM_012659	3.96	0.42	response to acid, hormone activity, extracellular region, extracellular space
Rdh7	retinol dehydrogenase 7	NM_133543	3.94	0.34	retinol dehydrogenase activity, binding
Slc15a1	solute carrier family 15 (oligopeptide transporter), member 1	NM_001079838	3.92	0.44	transporter activity
Slc27a2	solute carrier family 27 (fatty acid transporter), member 2	NM_031736	3.70	0.47	very long-chain fatty acid metabolic process
Prss35	protease, serine, 35	NM_001008560	3.61	0.32	catalytic activity, serine-type endopeptidase activity
Slc15a1	solute carrier family 15 (oligopeptide transporter), member 1	NM_001079838	3.30	0.40	transporter activity
Car7	carbonic anhydrase 7	NM_001106165	3.15	0.46	carbonate dehydratase activity
Adh4	alcohol dehydrogenase 4 (class II), pi polypeptide	NM_017270	3.11	0.20	retinoid metabolic process, respiratory system process, NADPH:quinone reductase activity

*Nineteen genes listed in above table were the things representing the high rate of change selected from 89 down regulation genes.

**Table 8:** List of genes (22) recovered by the AEtLP treatment among the total genes up-regulated by constipation.

GeneSymbol	GeneName	Accession No.	Fold of change in vehicle-treated group	Fold of change in AEtLP-treated group	GO category
RGD1311847	similar to 1700030K09Rik protein	NM_001013879	9.14	0.95	
Cyp2b2	cytochrome P450, family 2, subfamily b, polypeptide 2	NM_001198676	4.49	0.52	monooxygenase activity, endoplasmic reticulum
Ace	angiotensin I converting enzyme (peptidyl-dipeptidase A) 1	NM_012544	4.43	0.57	response to hypoxia, kidney development
Cyp2j4	cytochrome P450, family 2, subfamily j, polypeptide 4	NM_023025	2.96	0.63	retinoid metabolic process
G6pc	glucose-6-phosphatase, catalytic subunit	NM_013098	2.95	0.53	catalytic activity
Setbp1	SET binding protein 1	ENSRNOT00000021717	2.82	0.65	DNA binding
Nphs1	nephrosis 1 homolog (human)	NM_022628	2.73	0.57	MAPKKK cascade, membrane fraction
Map3k13	mitogen-activated protein kinase kinase kinase 13	BC081976	2.71	0.65	activation of MAPKK activity
Cmtm2a	CKLF-like MARVEL transmembrane domain containing 2A	NM_001013142	2.66	0.52	transcription corepressor activity, nucleus
LOC689709	similar to FERM domain containing 1	XM_001071738	2.49	0.62	binding, cytoskeleton
Treml1	triggering receptor expressed on myeloid cells-like 1	NM_001192001	2.49	1.30	cell surface, calcium-mediated signaling
Ptprr	protein tyrosine phosphatase, receptor type, R	NM_053594	2.45	0.51	in utero embryonic development, protein tyrosine phosphatase activity
Slc5a11	solute carrier family 5 (sodium/glucose cotransporter), member 11	NM_001100482	2.43	0.65	transporter activity, plasma membrane, ion transport
Raet1l	retinoic acid early transcript 1L	NM_001013063	2.32	0.73	immune response, membrane
Slc15a1	solute carrier family 15 (oligopeptide transporter), member 1	NM_057121	2.31	0.61	transporter activity
LOC689766	hypothetical protein LOC689766	NM_001109549	2.27	0.51	
Ghr	growth hormone receptor	NM_017094	2.14	0.71	activation of MAPK activity
Abcb9	ATP-binding cassette, subfamily B (MDR/TAP), member 9	NM_022238	2.12	0.53	nucleotide binding, protein transport, membrane
Lhfpl2	lipoma HMGIC fusion partner-like 2	NM_001106402	2.12	0.74	
Map3k13	mitogen-activated protein kinase kinase kinase 13	NM_001013978	2.07	0.77	activation of MAPKK activity
LOC689412	similar to CG4025-PA	NM_001109538	2.04	0.81	
Vav2	vav 2 guanine nucleotide exchange factor	NM_001106563	2.04	0.70	guanyl-nucleotide exchange factor activity, Rho guanyl-nucleotide exchange factor activity

To characterize the genes up-regulated by the AEtLP treatment after down-regulation induced by constipation, those showing at least a 2-fold change were selected from the ten up-regulated genes and five recovered genes. Of the ten up-regulated genes, the largest decrease in the transcripts of constipation rats was observed for Serpina3n (0.19-fold), followed Lcn2, Slc5a8, C3, Rerg, and Arhgap8 (Table 9). In addition, five up-regulated genes, including the Tmem45a gene, Rerg and Rgc32, were recovered to the normal levels after the AEtLP treatment (Table 10). Overall, these results suggest that the AEtLP treatment is closely involved in regulating the genes associated with transporters, metabolic enzymes, growth factor receptors, and blood vessel development under the condition of constipation induced by a loperamide treatment.

**Table 9:** List of genes (10) up-regulated by the AEtLP treatment among the total genes down-regulated by constipation.

GeneSymbol	GeneName	Accession No.	Fold of change in vehicle-treated group	Fold of change in AEtLP-treated group	GO category
Serpina3n	serine (or cysteine) peptidase inhibitor, clade A, member 3N	NM_031531	0.19	2.58	cell fraction, serine-type endopeptidase inhibitor activity
RGD1561738	similar to interferon induced transmembrane protein 2 (1-8D)	XR_008822	0.21	4.43	
Lcn2	lipocalin 2	NM_130741	0.23	3.48	protease binding, transporter activity
Slc5a8	solute carrier family 5 (iodide transporter), member 8	NM_001191987	0.25	2.84	transporter activity, plasma membrane, ion transport
C3	complement component 3	NM_016994	0.32	2.12	positive regulation of type IIa hypersensitivity
Rerg	RAS-like, estrogen-regulated, growth-inhibitor	XM_578417	0.34	2.06	GTPase activity, GTP binding, intracellular
Arhgap8	Rho GTPase activating protein 8	NM_001004242	0.38	2.33	intracellular, ignal transduction
Atrip	ATR interacting protein	NM_001106859	0.38	3.06	protein serine/threonine kinase activity
Serpina5	serine (or cysteine) peptidase inhibitor, clade A, member 5	NM_022957	0.39	3.18	protease binding
Tmem45a	transmembrane protein 45A	NM_001191074	0.39	2.20	

**Table 10:** List of genes (5) recovered by the AEtLP treatment among the total genes down-regulated by constipation.

GeneSymbol	GeneName	Accession No.	Fold of change in vehicle-treated group	Fold of change in AEtLP-treated group	GO category
Tmem45a	transmembrane protein 45A	NM_001191074	0.29	1.16	
RGD1563996	similar to Protein UNQ9166/PRO28631 precursor	ENSRNOT00000048111	0.33	1.72	
Rerg	RAS-like, estrogen-regulated, growth-inhibitor	XM_001072746	0.41	1.69	GTPase activity, GTP binding, intracellular
LOC688778	similar to fatty aldehyde dehydrogenase-like	ENSRNOT00000024034	0.43	1.21	aldehyde dehydrogenase [NAD(P)+] activity
Rgc32	response gene to complement 32	NM_054008	0.44	1.87	nucleus, cytoplasm, microtubule organizing center

### Validation of microarray data

**Fig 3:**
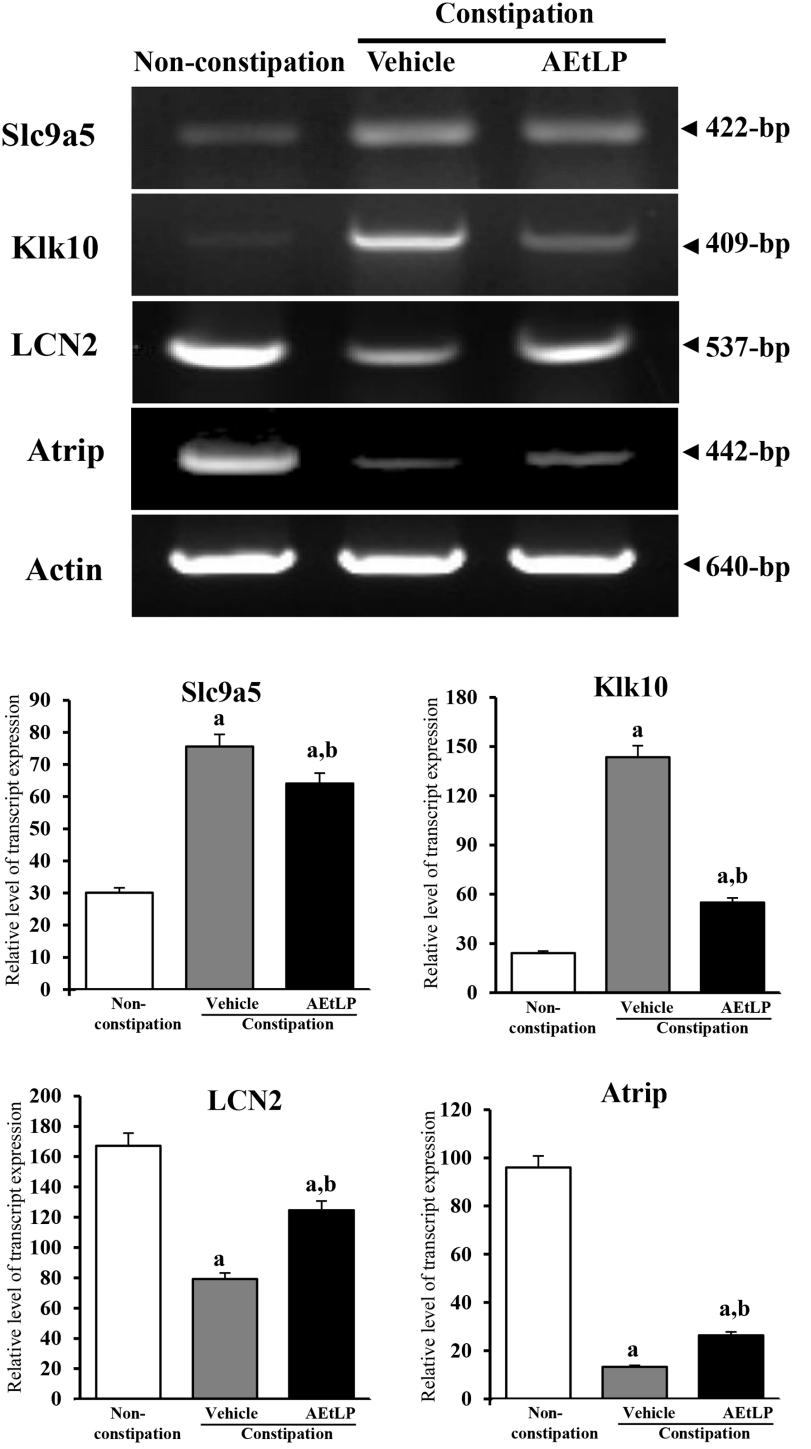
Change in the mRNA level of Slc9a5, Klk10, LCN2, and Atrip in non-constipation, vehicle- and AEtLP-treated constipation group. The distal colons of the rats in each group were pooled prior to mRNA analysis. The mRNA levels of the Slc9a5, Klk10, LCN2, and Atrip genes were examined by RT-PCR using the specific primers. The data shown are the means ± SD from three replicates. a, *P*<0.05 relative to the non-constipation group. b, *P*<0.05 relative to the vehicle-treated constipation group.

**Fig 4:**
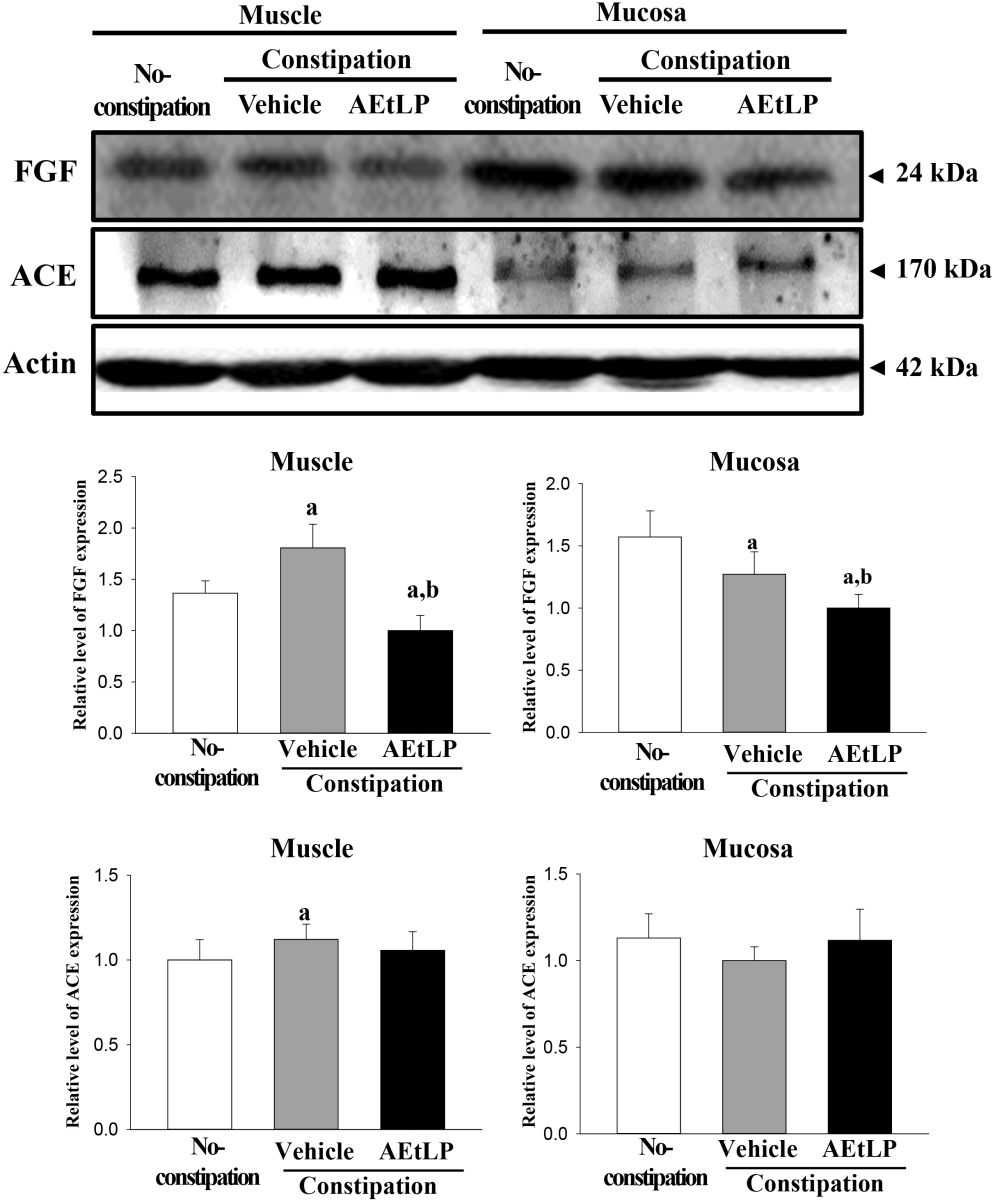
Change in the protein level of FGF and ACE in the muscle and mucosa layer of constipation, vehicle- and AEtLP-treated constipation group. Total tissue lysates were prepared from each layer collected from the distal colons of loperamide injected rats treated with vehicle or AEtLP as described in the Materials and Methods. A total of 50 μg of protein per sample were immunoblotted with specific antibodies for each protein. Three samples were assayed in triplicate by Western blotting. Data are reported as the mean±SD. a, *P*<0.05 relative to the non-constipation group. b, *P*<0.05 relative to the vehicle-treated constipation group.

To test the reliability of the results obtained from the microarray analysis, two up-regulated genes (Slc9a5, and Klk10) and two down-regulated genes (LCN2 and Atrip) were selected. When comparing the results of the microarray data and that of the RT-PCR, the pattern of increase was similar despite the difference in the increase or decrease rate (Fig 3). Furthermore, in the reliability test with western blot, the expression pattern of one down-regulated gene (Fgf) and one recovered gene (ACE) in muscle layer was consistent with the microarray data although the mucosa layer showed the different pattern (Fig 4). These results corroborate the data from microarray analysis. Especially, the protein expression from each layer indicated the possibility that the data from microarray may well reflect muscle layer than mucosa layer.

## Discussion

The expression of the genes in cells or an organism can be influenced by external signal factors, including natural products, drugs, chemicals, temperature, and light, as well as the organism's internal factors, including hormones and metabolism [20]. Among many external factors, this study focused on the correlation between phytochemical compounds and the improvement-related genes for constipation, even though many unknown substances remained in AEtLP. On the other hand, there are no reports of NHE5 and Klk10 altered by an AEtLP treatment correlating with a treatment of flavonoid and phenolic compounds, whereas accumulated studies have shown a correlation with other members within each gene family. In particular, the expression of three genes including LCN2, ACE2 and CYP2B2 is affected by the treatment of phytochemical compounds. The significant down-regulation of LCN2 mRNA was detected in ovarian cancer cells treated with estradiol and genistein, whereas the mRNA expression of ACE2 increased in the kidney after a high dose puerarin and apigenin treatment [21,22,23]. In addition, caffeic acid phenethyl ester and isoquercitrin induced a decrease in the CYP2B2 protein and mRNA level in the livers of Fischer 344 rats administered diethylnitrosamine [24,25]. Therefore, these results provide novel evidence that some genes listed in the present study may link the treatment of phytochemical compounds despite not showing a direct correlation between a single compound and each gene.

This study found several interesting genes related to the mechanism and pathogenesis of constipation. First, the Na^+^/H^+^ exchangers (NHE5) encoded in the Slc9a5 gene are membrane-bound transporters that catalyze the regulation of the intracellular pH and volume by mediating the electroneutral transport of H^+^ against the influx of Na^+^ ions [26]. NHE5 is expressed only in the brain and is tightly associated with end-stage renal disease and hypertension [27,28,29]. On the other hand, there are no reports indicating that NHE5 is a candidate protein for the regulation of constipation. In addition, decreased intestinal sodium absorption by selective NHE3 inhibition in the gut have been shown to induce a decrease in high blood pressure and an increase in feces water excretion [30]. In this study, the expression level of the NHE5 transcript was increased dramatically by a loperamide treatment, after which its level was recovered by the AEtLP treatment, as shown in Table 7. Accordingly, the results this study are the first to indicate NHE5 as key marker gene for screening therapeutic compounds with laxative effects. Nevertheless, further studies will be needed to determine the application possibility of NHE5 as a marker gene under clinical conditions.

Klk10 encodes serine proteases, including a member of the human tissue kallikrein family, and may play a role as a tumor suppressor gene to inhibit tumor formation in nude mice [31,32]. The exon 3 CpG island methylation of Klk10 is found in breast, ovarian, prostate, acute lymphoblastic leukemia (ALL), and gastric cancers [33,34]. Despite Klk10 being identified years ago as being prominently associated with cancer regulation, little is known about its biological involvement in intestinal diseases. Firstly, a relatively high level of active Klk was detected in the underlying colonic muscle of patients with ulcerative colitis, and diverticular disease [35]. In addition, a Klk inhibitor was considered to be a potential therapeutic agent for inflammatory arthritis and inflammatory bowel disease because the pretreatment of a specific plasma Klk inhibitor relieved the acute and chronic arthritis as well as the acute changes in the characteristics of enterocolitis [36]. In this study, the expression pattern of the Klk10 transcript was similar to that of the colonic muscle in patients with ulcerative colitis, even though their increase ratios differed between the two groups. Nevertheless, these results are the first to suggest that Klk10 is associated with the regulation of constipation in the loperamide and AEtLP-treated SD rats. Therefore, the above results including the present study suggest that an alteration of Klk expression may reflect an inflammatory condition induced by various factors in each tissue.

A large subset of cytochrome P450 (CYP) enzymes was expressed in various tissues including the liver, intestines, kidneys, and airways [37,38]. Among these subsets, the expression of CYP2B2 was induced by several structurally divergent compounds associated with phenobarbital, even though prototypical inducers, such as dexamethasone and pregnenolone 16α carbonitrile did not induce CYP2B2 expression [39]. On the other hand, a few studies have been conducted to determine if CYP2B2 expression can be regulated by a loperamide treatment. In the present study, the loperamide treatment induced an increase in CYP2B2 expression, but these levels were recovered to normal by the AEtLP treatment. On the other hand, further studies will be need to verify the possibility of an interaction between loperamide and AEtLP because the results from 128 case reports or case series and 80 clinical trials provided evidence for the possible interaction between herbal medicines and conventional drugs through induction analysis of CYP450 and or P-glycoprotein [40].

ACE is the major participant in the renin-angiotensin system (RAS), which regulates arterial vasoconstriction and the extracellular volume through the conversion of angiotensin I to angiotensin II [41,42]. ACE is also expressed in the lung, vascular endothelial cells, epithelial kidney cells, and testicular Leydig cells, but it is not expressed in the intestine [43]. Although no studies have examined the direct correlation between ACE and constipation, several accumulated reports suggest that the inhibition of ACE2 can ameliorate inflammatory bowel disease including ulcerative colitis and Crohn’s disease. The treatment of potent and selective AEC2 inhibitors, such as enalapril and GL1001, resulted in a markedly attenuated colon pathology and myeloperoxidase activity by down-regulating the NF-κB and TGF-β signaling pathway [44,45,46]. In the present study, an alteration of the regulation of ACE2 was similar to that of CYP2B2. As shown Table 7 and Fig 4, the AEtLP treatment induced recovery from the increased ACE mRNA and protein expression induced by the loperamide treatment. Especially, the significant alteration on this protein expression was detected in muscle layer. These findings provide novel information regarding the role of the ACE function in constipation regulation as well as the regulation of their expression in muscle layer of distal colon.

Lcn2 (lipocalin-2) is a 25-kDa secreted adipokine involved in the binding and transportation of small molecules, including retinoid, fatty acid, iron, and steroids [47,48,49,50,51]. This protein is associated with the pathogenesis of heart failure and kidney damage [52,53,54,55]. In particular, Lcn2 in microarray and proteomic analysis has been considered as a biomarkers of cancer diagnosis and drug efficacy in the colon of an azoxymethane-induced colon carcinogenesis model and pancreatic cancer of human patients [56,57]. Furthermore, this protein also marks the severity of intestinal inflammation because the significant up-regulation of Lcn2 also observed in both active ulcerative colitis and active Crohn’s disease [47,58]. Basically, a change in Lcn2 expression observed in the present work is an extension of previous studies, which indicated a role of the marker in the condition of several diseases. The present study provided novel evidence showing that Lcn2 can play a role as a sensitive and broad biomarker for constipation and laxative effects.

Fgf was widely known to multifunctional regulators affecting a variety of physiological regulations and events in mammals [59]. They contribute to organogenesis, tissue remodeling, nervous system regulation, angiogenesis and metabolism control [59,60,61]. Especially, Fgf-2 can regulate cell survival, proliferation, differentiation, migration, and replication of intestinal epithelial cells [62,63,64]. The protein level of Fgf-2 was increased in intestinal epithelial cells after radiation injury [65], while Fgf-19 expression in human ileum was induced by chenodeoxycholate and glycochenodeoxycholate treatment [66]. In this study, the expression of Fgf protein was increased or decrease in the muscle and mucosa layer of constipation rats induced with loperamide, although their level was simultaneously decreased after AEtLP treatment. Therefore, the present study provided first evidence that the expression of Fgf protein may differentially regulate in the muscle and mucosa layer of constipation rats.

Some extracts of *L*. *platyphylla* contain a wide variety of functional components, but further analyses will be needed to verify the correlation between their functions and therapeutic effects. Basically, the dry roots of *L*. *platyphylla* consist of carbohydrates (6.89 g/100 g) and sodium (Na; 6.32 g/100 g), and to a much lesser extent, proteins, fats, and sugars, whereas saturated fat, trans-fat and cholesterol are not present [14]. Moreover, AEtLP extracted from *L*. *platyphylla* roots contains saponins (1.73%), oligosaccharide (6.54%), succinic acid (111.48 mg), hydroxyproline (1,290 μg), and potassium (151.35 mg) [67]. In addition, the ethanolic extract of *L*. *platyphylla* was purified, leading to the isolation of five novel compounds including (+)platyphyllarin A/B, ethyltributanoate and (-)-liriopein A/B along with 21 known secondary metabolites [68]. Although the concentration and distribution of several components in *L*. *platyphylla* have been determined, further studies will be needed to examine the correlation between the components and expression of the corresponding genes.

Moreover, in humans, chronic constipation is subdivided into three groups based on an assessment of the colonic transit and anorectal function; 1) normal transit or irritable bowel syndrome, 2) pelvic floor dysfunction (functional defecatory disorders) and 3) slow transit constipation [69]. Indeed, more than 59% of patients with chronic constipation are normal transit. The remainder of the patients has functional defecatory disorders (25%), slow transit (13%) and a combination of defecatory disorders and slow transit (3%) [70]. Among these groups, models of pharmacological constipation induced by Lop can only show slow transit constipation, which presented less daily fecal excretion, lower water content, smaller number of fecal pellets and thinner fecal mucus [71, 72]. Therefore, these results have some limits and restrictions to a clinical translation of the results obtained from Lop-induced rats to human conditions, even though the experimental procedure for rat models, which is easy and reproducible without negative side effects or histologically detectable damage, is used widely for the evaluation and development of therapeutic drugs [73]. Further studies will be needed to determine the potential applications of NHE5, Klk10, CYP2B2, ACE2, and LCN2 as marker gene in clinical conditions.

In conclusion, the differential gene expression due to constipation and laxative effects was identified and validated using the transverse colon regions from vehicle- and AEtLP-treated constipation rats. The results of the present study identified 123-differentially expressed genes associated with constipation and laxative effects in chemically-induced constipation model rats, which were confirmed by RT-PCR. Therefore, these genes might be useful for the development of new therapeutic targets.

## Materials and Methods

### Analysis of bioactive compounds in the roots of LP

The amount of total phenolics in LP was determined using the Folin-Ciocalteu method [74]. The amount of total flavonoids in the RLP extract was determined according to the method reported by Meda et al. [75]. The concentration of spicatoside A and sugars in LP were analyzed using an iLC 3000 HPLC system (Interface Engineering, Seoul, Korea) equipped with a Corona CAD Detector (ESA Bioscinece, Inc., Chelmsford, MA, USA). Chromatographic separation was performed on a CAPCELL PAK MG C18 (4.6 mm×250 mm, particle size 5 μm, Shiseido Co. Ltd., Tokyo, Japan).

### Induction of constipation and animal experimental design

The animal protocol used in this study was reviewed and approved based on the ethical procedures and scientific care by the Pusan National University-Institutional Animal Care and Use Committee (Approval Number; PNU-2012-0010). All animals were handled at the Pusan National University Laboratory Animal Resources Center accredited by AAALAC International (Accredited Unit Number; 001525) according to the National Institutes of Health guidelines.

For the experiment, 8-week-old Sprague Dawley (SD) rats (n = 18) were assigned to either a non-constipation group (n = 6) or a constipation group (n = 12). Constipation was induced in the SD rats by a subcutaneous injection of loperamide (4 mg/kg weight) in 0.9% sodium chloride twice (9 AM and 6 PM) a day for 3 days, whereas the non-constipation group was injected with 0.9% sodium chloride alone, as described in a previous study [19]. At 15 hr after the final loperamide treatment, the constipation group was divided further into a vehicle-treated constipation group (n = 6) and AEtLP-treated constipation group (n = 6). They received a consistent volume of water or 15 μL/g body weight of AEtLP (1,000 mg/kg weight) via oral administration once at 9 AM. At 24 h after the AEtLP or vehicle treatments, all animals were euthanatized using CO_2_ gas and tissue samples were acquired and stored in Eppendorf tubes at -70°C until assayed. Furthermore, the stools and urine excreted from each SD rat were measured at 10:00 am every day in the metabolic cage (Daejong Co., Korea).

### Measurement of stool number and urinary volume

All SD rats were bred in metabolic cages during the experimental period to avoid contamination with other dust. The stools and urine excreted from each SD rat were collected at 9 AM every day during the experimental process in the metabolic cage (Daejong Co., Korea). The number of stools was counted using a key cell counter and the urine volume was measured three times using a measuring cylinder.

### Histological analysis

The distal colons collected from the SD rats were fixed with 10% formalin for 48 h, embedded in paraffin wax, sectioned into 4 μm thick slices, and then stained with hematoxylin & eosin (H&E, Sigma-Aldrich, MO, USA). The morphological features of these sections were observed by optical microscopy. The muscle thickness, flat luminal surface thickness, number of goblet cells, and number of lieberkuhn crypt were measured using the Leica Application Suite (Leica Mycrosystems, Switzerland).

For mucin staining, transverse colons collected from the rats were fixed with 10% formalin for 48 h, embedded in paraffin wax, and sectioned into 3 μm thick slices that were then deparaffinized with xylene and rehydrated. The samples were then rinsed with distilled water and stained using an Alcian blue stain kit (IHC WORLD, MD, USA). Finally, the stained colon sections were observed by optical microscopy and the size, number, and morphology of the crypts were measured using Leica Application Suite (Leica Microsystems, Switzerland).

For TEM analysis, the transverse colons collected from rats were first fixed in 2.5% glutaraldehyde in 1x PBS buffer, washed, dehydrated with ascending concentrations of ethanol, incubated in 1% OsO_4_ for 1 h at room temperature, and then embedded in Epon812 media (Polysciences, Inc., Germany). Ultra-thin sections (70 nm) were then collected on holy formvar coated grids, contrasted with uranyl acetate and lead citrate, and examined by TEM (Hitachi, Japan).

### RNA isolation

The distal colons from SD rats of the No, vehicle, and AEtLP treated groups were used to isolate the total RNA using Trizol (Invitrogen Life Technologies, Carlsbad, USA), after which they were purified using RNeasy columns (Qiagen, Valencia, USA). Both procedures were conducted according to the manufacturer’s protocol. Briefly, 100 mg of the frozen distal colons were homogenized in a lysis buffer using a rotor-stator homogenizer. Subsequently, 70% ethanol was added to the homogenate, after which the samples were applied to an RNAeasy minispin column to bind the total RNA, which was then eluted in RNase-free-water. After DNase digestion and clean-up, the RNA samples were quantified, divided into aliquots and stored at -80°C until needed. For quality control, the RNA purity and integrity were evaluated by denaturing gel electrophoresis, OD 260/280 ratio, and analysis on an Agilent 2100 Bioanalyzer (Agilent Technologies, Palo Alto, USA).

### Labeling and purification

The total RNA was amplified and purified using an Ambion Illumina RNA amplification kit (Ambion, Austin, USA). To produce biotinylated cRNA according to the manufacturer’s instructions, 550 ng of the total RNA was reverse-transcribed to cDNA using a T7 oligo(dT) primer. A second-strand of cDNA was synthesized, transcribed *in vitro*, and labeled with biotin-NTP. After purification, the cRNA was quantified using an ND-1000 Spectrophotometer (NanoDrop, Wilmington, USA).

### Hybridization and data export

The labeled cRNA samples (750 ng) were hybridized to each Rat-6 Expression BeadChip for 16–18 h at 58°C according to the manufacturer's instructions (Illumina, Inc., San Diego, USA). The array signal was then detected using Amersham fluorolink streptavidin-Cy3 (GE Healthcare Bio-Sciences, Little Chalfont, UK) according to the manufacturer's protocols. The arrays were scanned using an Illumina bead array reader confocal scanner (BeadStation 500GXDW; Illumina, Inc., San Diego, CA) according to the manufacturer's instructions. The array data export processing and analysis was performed using an Illumina BeadStudio.

### Raw data preparation

The quality of hybridization and the overall chip performance were monitored by a visual inspection of both the internal quality control checks and the raw scanned data. The raw data was extracted using the software provided by the manufacturer (BeadStudio v.3.1). The array data was filtered using a *P* value set at <0.05 in at least 50% of the samples. A filtering criterion was applied for data analysis, with higher signal values required to obtain a detection (*P* <0.05). The selected gene signal values were log transformed and normalized using the quantile method to remove systemic bias. The data reliability was measured using the scatter plot method. The correlation coefficients (Rs) of the normalized samples ranged from 0.95 to 0.99, which indicates that a systematic variation of non-biological origin was removed. Comparative analysis between the vehicle-treated rats and AEtLP-treated rats samples was conducted using a *t*-test (|fold|> or <2) and the adjusted Benjamini-Hochberg FDR (false discovery rate) (*P* <0.05). Also, if the value of fold change are between 0.5< and <1.5 after AEtLP treatment, these gene decided as recovered genes and now functioning normally. Furthermore, volcano plots and hierarchical cluster analysis were conducted using a complete linkage and Euclidean distance as a measure of similarity. Time-dependent profiling was achieved by k-means cluster analysis (k; 9, Euclidean distance, complete linkage). All data analysis and visualization of the differentially expressed genes was conducted using ArrayAssist (Stratagene, La Jolla, USA). The biological pathway and ontology-based analysis were performed using the PANTHER (Protein ANalysis THrough Evolutionary Relationships) database (http://www.pantherdb.org). All microarray data was submitted to the GEO database (http://www.ncbi.nih.gov/geo) under accession number GSE62041.

### RT-PCR

The complementary DNA was synthesized using mixture containing the total RNA (5 μg), oligo-dT primer (500 ng, Invitrogen, CA, USA), dNTP and reverse transcriptase (200 units, Invitrogen) according to the method described elsewhere [76]. Furthermore, the PCR product of each gene was amplified using the specific primers, as described in previous studies [77].

### Western blot

Firstly, in order to separately collect muscle and mucosa layer, the distal colon of SD rats were cut along a colon tube using scissors and washed with 1x PBS. Next, the mucosa layer were scraped out from the muscle layer using surgical knife and collected in homogenized tube. Whole distal colon, muscle layer and mucosa layer collected from a subset of the groups were homogenized using a PRO-PREP Solution Kit (iNtRON Biotechnology, Sungnam, Korea) supplemented with half of a protein inhibitor cocktail tablet (Roche, Penzberg, Germany), then centrifuged at 10,000 ×g for 10 min. The prepared proteins were subsequently subjected to 10% SDS-PAGE, after which they were transferred to a nitrocellulose membrane (Amersham Biosciences, Corston, UK) for 2 h at 45 V in transfer buffer (25 mM Trizma-base, 192 mM glycine, and 20% methanol). The efficiency of the transfer and equal protein loading were evaluated by staining the membrane with Amido Black Staining Solution (Sigma-Aldrich Co.) and the gel with Coomassie Blue. Appropriate dilutions of primary antibodies, anti-FGF antibody (Santa Cruz, CA, USA), anti-ACE antibody (R&D Systems, Minneapolis, MN, USA) and anti-β-actin (Sigma-Aldrich Co.) were added to the membranes and allowed to hybridize overnight at 4°C. After the antibodies were removed, the membrane was washed three times in a solution composed of 10 mM Trizma-base (pH 7.6), 150 mM NaCl, and 0.05% Tween-20 for 10 min. The membrane was then incubated with horseradish peroxidase-conjugated anti-secondary antibody for 1 h at room temperature, after which it was washed again as described above and developed using an enhanced chemiluminescence detection system (Amersham Biosciences). Finally, the results were quantified using the Image Analyzer System (Eastman Kodak 2000MM, Eastman Kodak, Rochester, NY, USA) and expressed as the fold-increase over control values. These results were confirmed by two independent researchers conducting the experiments at least twice.

### Statistical analysis

The significant differences in the results of microarray analyses between the non-constipation group and constipation group were identified using a *t-test*. In addition, one-way ANOVA (SPSS for Windows, Release 10.10, Standard Version, Chicago, IL) was used to determine the variance and to identify the significant differences in the specific mRNA expression between the vehicle-treated constipation group and AEtLP-treated constipation group. All values are presented as the mean ± standard deviation (SD). A *P* <0.05 was considered significant.
